# Mycorrhizal Fungal Effects on Plant Growth, Osmolytes, and *CsHsp70s* and *CsPIPs* Expression in Leaves of Cucumber under a Short-Term Heat Stress

**DOI:** 10.3390/plants12162917

**Published:** 2023-08-11

**Authors:** Xin-Ran Liu, Zi-Yi Rong, Xiao Tian, Abeer Hashem, Elsayed Fathi Abd_Allah, Ying-Ning Zou, Qiang-Sheng Wu

**Affiliations:** 1 College of Horticulture and Gardening, Yangtze University, Jingzhou 434025, China; 15275809238@163.com (X.-R.L.);; 2 Botany and Microbiology Department, College of Science, King Saud University, P.O. Box 2460, Riyadh 11451, Saudi Arabia; 3 Plant Production Department, College of Food and Agricultural Sciences, King Saud University, P.O. Box 2460, Riyadh 11451, Saudi Arabia

**Keywords:** aquaporin, arbuscular mycorrhizal fungi, cucumber, heat shock protein, osmotic adjustment

## Abstract

Arbuscular mycorrhizal (AM) fungi enhance plant stress tolerance, but it is unclear whether AM fungi affect heat tolerance in cucumbers. This study aimed to analyze how an AM fungus, *Diversispora versiformis*, affected growth, chlorophyll, five osmolytes, and plasma membrane intrinsic proteins (*PIPs*) and heat shock protein 70 (*Hsp70*) gene expression in cucumber leaves after a short-term (80 h) heat stress. Heat treatment significantly reduced root AM fungal colonization rate (0.26 folds). Heat treatment also distinctly suppressed plant height, stem diameter, and biomass, whereas AM fungal inoculation improved these growth variables as well as the chlorophyll index, with the benefit being more obvious under heat than under no-heat stress conditions. Heat treatment triggered differential changes in osmolytes (sucrose, fructose, and betaine) of inoculated and uninoculated cucumbers, whereas inoculation with AM fungus significantly raised leaf sucrose, fructose, glucose, betaine, and proline levels when compared to non-AM fungal inoculation. Heat treatment increased the expression of two (*CsPIP1;6* and *CsPIP2;1*) of eight *CsPIPs* in inoculated and uninoculated plants, whereas AM fungal inoculation up-regulated the expression of *CsPIP1;6*, *CsPIP2;1*, and *CsPIP2;6* under heat stress conditions. *Hsp70s* expressed differently in inoculated and uninoculated plants under heat versus no-heat stress, with 6 of 11 *CsHsp70s* down-regulated in inoculated plants. Under heat stress conditions, AM fungus only up-regulated *CsHsp70-8* expression in 11 *Hsp70s*, while another eight *CsHsp70s* were down-regulated. Heat treatment and AM fungal inoculation both increased the expression of *CsHsp70-8* and *CsPIP1;6*. It was concluded that AM fungus-inoculated cucumbers have high levels of growth, chlorophyll, and osmolytes under heat stress and do not require high *CsPIPs* and *CsHsp70s* expression to tolerate a short-term heat treatment.

## 1. Introduction

Cucumber (*Cucumis sativus* L.) is a popular vegetable crop worldwide with a warm but thermosensitive growth habit [1]. Cucumber plants grow best at temperatures of 25–30 °C/13–15 °C (day/night temperature) [2]. When the ambient temperature exceeds 35 °C, cucumbers suffer heat damage, resulting in reduced plant growth, low pollination success, and fruit abnormalities; at 50 °C, cucumber leaves and stems can soon wilt and die [3]. Therefore, heat (H) stress is the main stress element in cucumber cultivation during the summer, and it is critical to improve cucumber’s H resistance.

Plant-growth-promoting microorganisms have shown strong potential to enhance plant stress resistance and, thus, promote sustainable agricultural development [4,5,6,7]. Plant rhizospheres are known to be inhabited by arbuscular mycorrhizal (AM) fungi that establish reciprocal symbionts with their roots [8]. Arbuscular mycorrhizae in roots aid the uptake of soil water and nutrients by the host plants, while the plants provide sugars and lipids to meet the energy requirements of AM fungi [9]. AM fungi are important for enhancing the resistance of host plants to various abiotic stresses [10]. Therefore, AM fungi are seen as an environmentally acceptable method for combating abiotic stresses in vegetable crops, together with reduced inorganic fertilizer use [11,12]. Mycorrhizal maize plants had higher leaf gas exchange, resulting in an increase in plant biomass and photosynthate under H stress (44 ± 0.2 °C) conditions, compared with non-mycorrhizal plants [13]. Reva et al. [14] introduced *Rhizophagus irregularis* into tomato, pepper, and cucumber plants and found that AM fungal inoculation increased their growth vigor, productivity, and fruit quality under H stress conditions, thus improving the endurance to H stress. *Glomus fasciculatum* alleviated H damage in cyclamen plants by increasing antioxidant enzyme activities at 30 °C during a four-week period [15]. In wheat, AM fungal inoculation also increased grain number and regulated nutrient composition, with a low potassium/calcium ratio [16]. In tomato, the expression of root *9-cis-epoxycarotenoid dioxygenase* and *plasma membrane intrinsic protein 2.7* genes was not affected by AM fungal inoculation under drought + heat stress conditions, while *lipoxygenase D* gene expression was increased by *Septoglomus constrictum*, but not *Septoglomus deserticola* under drought + heat stress conditions [17]. These findings demonstrated that AM fungi could enhance the H tolerance of host plants, but the underlying mechanism remains unknown, particularly for stressed genes. In addition, the research on the effect of AM fungi on the H resistance of host plants is relatively slow, relative to the research on the mechanism of AM fungi in resistance to drought and salt stress [11].

AM fungi have been applied to cucumber plants and have been shown to enhance cucumber resistance to soil-borne pathogens (e.g., *Rhizoctonia solan*), salt stress, and low-temperature stress [18,19,20]. However, it is not clear whether and how AM fungi enhance the tolerance of a short-term H in cucumbers. Since AM fungi have shown good H resistance in other plants, we hypothesized that AM fungi could enhance the H tolerance in cucumbers, associated with the improvement of physiological activities and the up-regulated expression of stress-responsive genes. The aim of this study was to investigate the effects of an AM fungus on growth, chlorophyll index, organic solute levels, and the expression levels of two stress-responsive gene (plasma membrane intrinsic proteins, *PIPs*; heat shock protein 70, *Hsp70*) family members under a short-term H (80 h) stress.

## 2. Results

### 2.1. Changes in Root AM Fungal Colonization

The mycorrhizal fungus *D. versiformis* successfully colonized cucumber roots with root colonization rates of 47.31% to 64.10% (Table 1). The H treatment significantly inhibited mycorrhizal colonization rates by 26.19%, compared with the no heat stress (NH) treatment.

### 2.2. Changes in Growth Behavior

H treatment also significantly inhibited plant height, stem diameter, and biomass by 19.09%, 15.19%, and 10.81% in inoculated plants and by 41.46%, 18.18%, and 44.62% in uninoculated plants, respectively, showing that the inhibitive magnitude was greater in uninoculated plants than in inoculated plants (Table 1). On the other hand, inoculation with AM fungi significantly increased plant height, stem diameter, and biomass by 168.29%, 62.20%, and 205.88% under NH conditions as well as by 270.83%, 68.13%, and 392.67% under H conditions, respectively.

### 2.3. Changes in Chlorophyll Index

**Figure 1:**
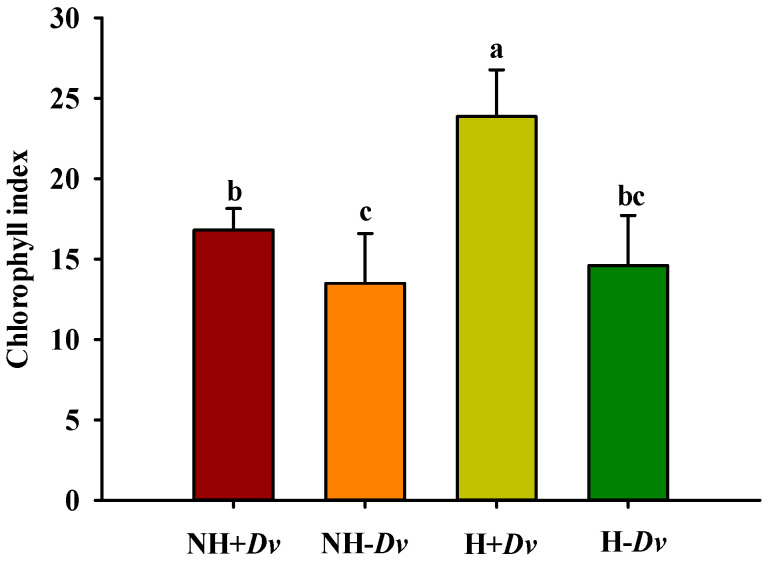
AM fungal effects on leaf chlorophyll index of cucumbers under no heat stress and heat stress conditions. Data (means ± SD, *n* = 5) followed by different letters above the bars indicate significant (*p* < 0.05) differences among treatments. See Table 1 for abbreviations.

H stress obviously altered leaf chlorophyll index, especially in inoculated plants, where chlorophyll index was significantly increased by 42.08%, compared with NH treatment (Figure 1). AM fungal inoculation significantly increased chlorophyll index by 24.44% and 63.49% under NH and H conditions, respectively.

### 2.4. Changes in Sugar Levels

**Figure 2:**
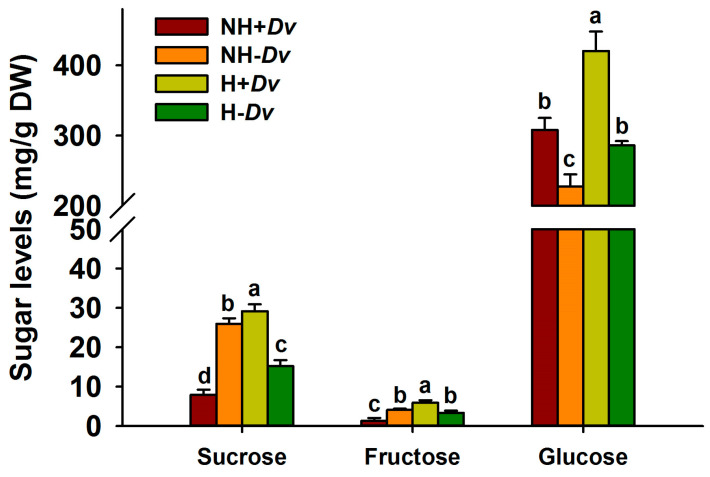
AM fungal effects on leaf sucrose, fructose, and glucose levels of cucumbers under no heat stress and heat stress conditions. Data (means ± SD, *n* = 4) followed by different letters above the bars indicate significant (*p* < 0.05) differences among treatments. See Table 1 for abbreviations.

The concentration of sugars in leaves was dependent on temperature treatments and inoculations (Figure 2). In uninoculated plants, H treatment significantly decreased leaf sucrose levels by 41.44%, but it distinctly increased leaf glucose levels by 25.84%, with no significant effect on fructose, compared with NH treatment. In inoculated plants, however, H treatment significantly increased leaf sucrose, glucose, and fructose levels by 268.14%, 334.56%, and 36.55%, respectively, in comparison with NH treatment. In addition, AM fungal inoculation significantly increased leaf glucose levels under NH conditions by 35.53%, and it also decreased the levels of sucrose and fructose by 69.61% and 66.67%, respectively, compared with non-AM treatment. Nevertheless, AM fungi significantly increased the levels of sucrose, fructose, and glucose by 91.71%, 77.48%, and 47.06%, respectively, compared with the uninoculated treatment.

### 2.5. Changes in Betaine and Proline Levels

**Figure 3:**
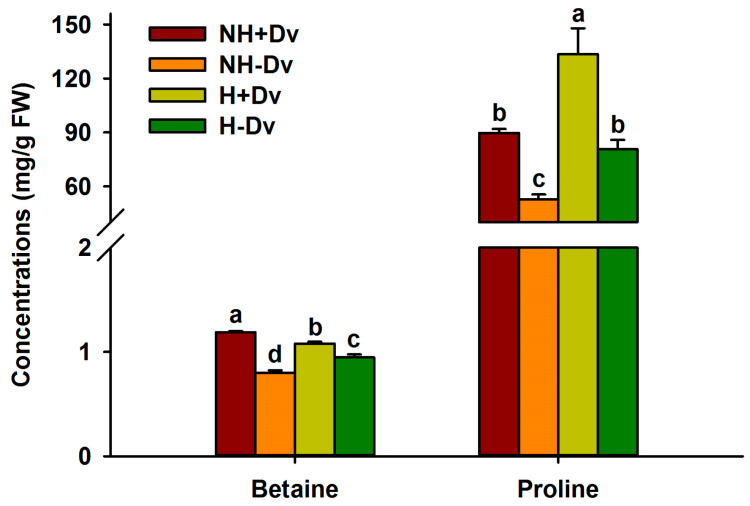
AM fungal effects on leaf betaine and proline levels of cucumbers under no heat stress and heat stress conditions. Data (means ± SD, *n* = 4) followed by different letters above the bars indicate significant (*p* < 0.05) differences among treatments. See Table 1 for abbreviations.

Compared to NH treatment, H treatment significantly inhibited betaine levels in inoculated plants by 9.17%, but increased proline levels by 48.67%; in uninoculated plants, H treatment significantly increased betaine and proline levels by 18.24% and 53.24%, respectively (Figure 3). Moreover, when cucumber plants were inoculated with *D*. *versiformis*, betaine and proline levels were significantly increased by 48.33% and 70.37% under NH conditions and by 13.94% and 65.30% under H conditions, respectively. 

### 2.6. Changes in Leaf CsPIPs Expression

**Figure 4:**
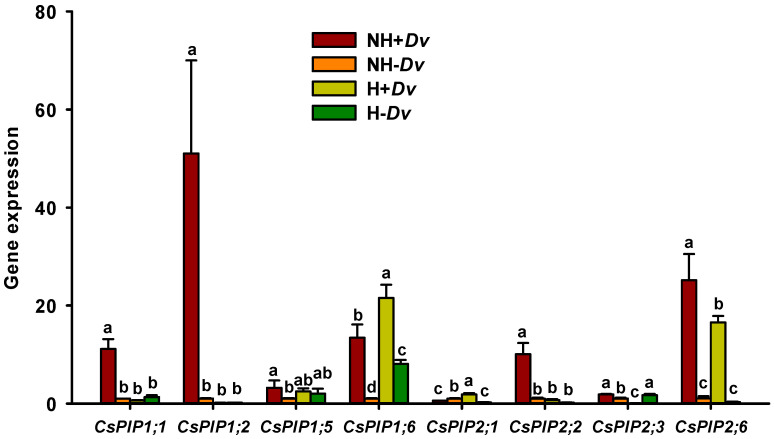
AM fungal effects on the expression of eight *CsPIPs* of cucumber leaves under no heat stress and heat stress conditions. Data (means ± SD, *n* = 3) followed by different letters above the bars indicate significant (*p* < 0.05) differences among treatments. See Table 1 for abbreviations.

In inoculated plants, the H treatment significantly induced an up-regulation of *CsPIP1;6* and *CsPIP2;1* expression by 0.60- and 2.16-fold, respectively, and it down-regulated the expression of *CsPIP1;1*, *CsPIP1;2, CsPIP2;2*, *CsPIP2;3*, and *CsPIP2;4* by 0.94-, 1.00-, 0.93-, 0.95-, and 0.34-fold, respectively, plus no significant changes in *CsPIP1;5* expression, compared with the NH treatment (Figure 4). In uninoculated plants, the H treatment significantly up-regulated *CsPIP1;6* and *CsPIP2;3* expression by 7.08- and 0.70-fold, respectively, compared to the NH treatment, with a down-regulated effect on *CsPIP2;1* expression (0.77-fold) and no significant changes in other *CsPIPs*. In addition, inoculation with AM fungus also triggered diverse changes in *CsPIPs* expression: under NH conditions, *CsPIP1;1*, *CsPIP1;2*, *CsPIP1;5*, *CsPIP1;6*, *CsPIP2;2*, *CsPIP2;3*, and *CsPIP2;6* were up-regulated by 10.14-, 49.93-, 2.21-, 12.40-, 8.93-, 0.83-, and 22.42-fold, respectively, but *CsPIP2;1* was down-regulated by 0.41-fold; under H condition, *CsPIP1;6*, *CsPIP2;1*, and *CsPIP2;6* were up-regulated by 1.66-, 6.94-, and 50.69-fold, respectively, and *CsPIP2;3* was down-regulated by 0.95-fold.

### 2.7. Changes in Leaf CsHsp70s Expression

**Figure 5:**
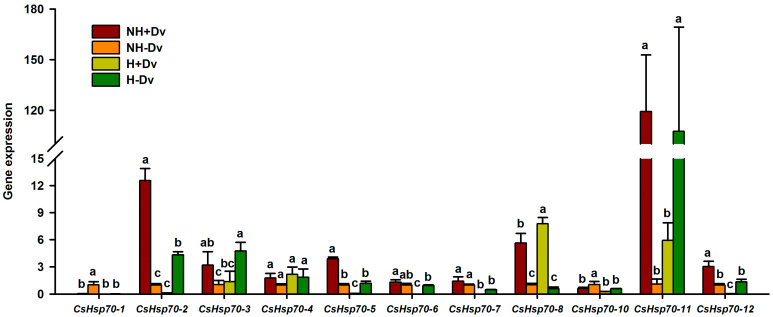
AM fungal effects on the expression of eleven *CsHsp70s* of cucumber leaves under no heat stress and heat stress conditions. Data (means ± SD, *n* = 3) followed by different letters above the bars indicate significant (*p* < 0.05) differences among treatments. See Table 1 for abbreviations.

*CsHsp70s* genes showed diverse expression patterns in the inoculated and uninoculated plants (Figure 5). In mycorrhizal plants, H treatment only induced up-regulated expression of *CsHsp70-8* by 0.38-fold, while it down-regulated expression of *CsHsp70-2*, *CsHsp70-5*, *CsHsp70-6*, *CsHsp70-7*, *CsHsp70-11*, and *CsHsp70-12* by 0.99-, 0.98-, 0.98-, 0.99-, 0.95-, and 0.99-fold, respectively, as compared with NH treatment. Similarly, in non-mycorrhizal plants, H treatment up-regulated the expression of *CsHsp70-2*, *CsHsp70-3*, and *CsHsp70-11*, each by 3.28-, 3.46-, and 96.40-fold, but also down-regulated the expression of *CsHsp70-1*, *CsHsp70-7*, and *CsHsp70-10*, each by 0.96-, 0.52-, and 0.43-fold.

The effect of AM fungal inoculation on the expression of *CsHsp70s* also varied according to the stress and *CsHsp70* homologs (Figure 5). Under NH conditions, AM fungal inoculation significantly increased *CsHsp70-2*, *CsHsp70-3*, *CsHsp70-5*, *CsHsp70-8*, *CsHsp70-11*, and *CsHsp70-12* expression by 11.48-, 2.00-, 2.86-, 4.56-, 107.02-, and 2.01-fold, respectively, whereas it inhibited the expression of *CsHsp70-1* and *CsHsp70-10* by 0.96- and 0.40-fold, respectively, compared with non-AM treatment. Similarly, under H conditions, AM fungus only induced up-regulated expression of *CsHsp70-8* by 12.13-fold, whereas *CsHsp70-2*, *CsHsp70-3*, *CsHsp70-5*, *CsHsp70-6*, *CsHsp70-8*, *CsHsp70-9*, *CsHsp70-11*, and *CsHsp70-12* expression was down-regulated by AM fungal inoculation by 0.97-, 0.71-, 0.94-, 0.98-, 0.94-, and 0.98-fold, respectively, compared with non-AM treatment.

## 3. Discussion

In the present study, an 80 h heat treatment considerably suppressed AM fungal colonization rate in cucumber roots, which is consistent with Zhu et al. [21] in maize inoculated with *Glomus etunicatum* at 40 °C for one week. Temperature is an important factor affecting spore germination of AM fungi and root AM fungal colonization of host plants, as shown by the fact that both low and high temperatures inhibit root AM fungal colonization degree [18,20,21,22,23]. In *Cyclamen persicum* plants, a 4-week heat treatment (30 °C) did not affect the root colonization by *G. fasciculatum* [15]. This implies that the effects of H stress on root AM fungal colonization are dependent on the stress temperature and stress duration. Generally, root AM fungal colonization decreases at temperatures above 30 °C, and temperatures above 40 °C are lethal to AM fungi [24].

The present study also observed that the growth of cucumber seedlings was inhibited by the H stress, and the inhibitory effect was greater in uninoculated plants than in inoculated plants. This indicates that the inoculated plants received a relatively smaller effect of H stress than the uninoculated plants. It has been documented that H stress can strongly inhibit plant growth, including cucumber seedlings [25,26]. AM fungal inoculation also significantly increased plant height, stem diameter, and biomass of cucumbers, and this increase was more pronounced under H conditions than under NH conditions. As a result, AM fungus-inoculated cucumbers had greater adaptability to H treatment than uninoculated plants. Reva et al. [14] also reported that inoculation with *Rhizophagus irregularis* significantly improved the growth of both tomato and pepper under H treatment. As a result, AM fungal inoculation had positive effects on plant growth behavior of cucumbers under H conditions, suggesting the potential of arbuscular mycorrhizae to enhance the tolerance of H stress in cucumbers.

In our study, the H treatment promoted glucose levels but suppressed sucrose levels in cucumber leaves, along with an increase in fructose levels in inoculated plants, compared with the NH treatment. This change is linked to the fact that the H treatment promotes cleavage of sucrose to hexose, especially glucose [27]. AM fungal inoculation distinctly raised glucose, fructose, and sucrose levels in cucumber leaves under H stress, which is consistent with the results of Zhang et al. [28], who inoculated trifoliate orange seedlings with AM fungi under drought stress. Under unfavorable temperature stress, plants usually undergo biochemical changes, particularly by increasing compatible solutes to enhance osmotic adjustment [29]. In osmolytes, soluble sugars, proline, and betaine are involved in maintaining osmotic balance to resist unfavorable conditions [30]. Thus, the high accumulation of soluble sugars in mycorrhizal plants provides protection to cell membrane integrity as well as osmoregulation, thus recording a higher stress defense response in mycorrhizal plants than in non-mycorrhizal plants under a short-term H stress.

Our study showed that both proline and betaine levels in cucumber leaves were significantly increased by AM fungal inoculation under both NH and H conditions. This implies that mycorrhizal cucumbers respond to H stress by accumulating betaine and proline. This is in agreement with a previous work on flooded peach trees inoculated with AM fungi to obtain proline elevation [31] and in NaCl citrus seedlings, where betaine was elevated by AM fungi [32]. Ali et al. [33] also confirmed that H-stress-tolerant cucumber varieties presented higher proline than H stress sensitive vouchers. However, Zhu et al. [21], on the other hand, found in maize that AM fungal treatment at high temperature significantly reduced proline content but had no effect on root proline. This shows that mycorrhizal-regulated proline changes under H stress are host tissue-specific. Betaine and proline are important osmoregulatory substances involved in osmotic adjustment, where betaine also stabilizes the structure of enzymes and the integrity of the plasma membrane, while proline is also a scavenger of free radicals as well as a molecular chaperone in redox homeostasis [30]. Therefore, the presence of higher proline and betaine levels in mycorrhizal versus non-mycorrhizal cucumber plants under H conditions suggests that mycorrhizal plants have a stronger ability of osmotic adjustment to adapt H stress for maintaining cell water status and protecting the denaturation of membrane and proteins. In addition, plant growth promoting microorganisms regulate proline levels in plants through modulating proline metabolism (e.g., P5CS, P5CR, and PDH) [6]. It remains to be clarified whether the mechanism occurred in this study. 

Aquaporins are transmembrane transporters that play an important role in plant defense responses to biotic and abiotic stresses, where *PIPs* are transporters of carbon dioxide, glycerol, peroxide hydrogen, urea, and water [34]. This study indicated that the expression of *CsPIPs* in inoculated and uninoculated cucumbers was different under H versus NH conditions, where *CsPIP1;6* and *CsPIP2;1* expression was increased in inoculated plants, and *CsPIP1;6* and *CsPIP2;3* were up-regulated in uninoculated plants. Zhu et al. [35] also found that four *CsPIPs* including *CsPIP2;1* were induced to be up-regulated by H treatment. Liu et al. [36] also observed differences in the expression of *PIPs* in AM- and non-AM tomato plants under salt stress versus non-salt stress conditions. In addition, AM fungi also triggered diverse changes in the expression of *CsPIPs*: seven out of eight *CsPIPs* were up-regulated by AM fungi under NH conditions, whereas only three *CsPIPs* were up-regulated under H conditions. Among them, both *CsPIP1;6* and *CsPIP2;6* were up-regulated by AM fungi under both NH and H conditions, showing high AM fungal specificity. Similarly, AM fungal inoculation of trifoliate orange and tomato plants triggered diverse regulation of aquaporin family members [37,38], and AM fungi made strategic changes in aquaporin gene expression depending on the type of environmental stress [39]. Qian et al. [40] found that *CsPIP2;6* contributed 2.8 ± 0.6% of the 10 *CsPIPs* expression, with an osmotic water permeability of 0.8 × 10^−2^ cm/s. This showed that *CsPIP2;6* contributed to water in mycorrhizal cucumber plants subjected to H stress, which facilitated the enhancement of plant stress resistance. However, *CsPIP1;6* was the only gene that was up-regulated by H stress and AM fungal inoculation. Although there is no evidence for the function of *PIP1;6* in cucumbers, it has been shown in maize and sorghum that *PIP1;6* was most likely involved in leaf CO_2_ transport [41]. Therefore, AM-modulated leaf *CsPIP1;6* expression may be involved in photosynthesis, but further studies are needed. Similarly, it is also necessary to determine how much the AM-regulated *CsPIPs* contribute to host water absorption.

The results of this study showed that among the 11 *Hsf70s* tested, only *CsHsp70-8* was up-regulated in inoculated plants under H versus NH conditions, and another six *CsHsps* genes were down-regulated. Nevertheless, *CsHsp70-2*, *CsHsp70-3*, and *CsHsp70-11* were up-regulated in uninoculated plants under H versus NH conditions. Similarly, under H conditions, AM fungi only up-regulated the expression of *CsHsp70-8* in the 11 *Hsf70* members, while other eight *CsHsf70s* were down-regulated. Plants are able to induce the expression of *Hsp* genes to adapt to H stress [42]. These highly conserved Hsp family members have molecular chaperone-like functions, and are also involved in signal transduction during H stress, with *Hsp70* being the most abundant and well-characterized protein family in eukaryotic cells [43]. Most *Hsfs* have strong cytoprotective roles, mainly preventing protein aggregation and facilitating protein folding [44]. Rivera-Becerril et al. [45] reported that inoculation with *Glomus intraradices* increased the *hsp70* expression in *Pisum sativum* roots under no-Cd stress, but this did not occur under Cd stress. Furthermore, *CsHsp70-8* expression was up-regulated by both H treatment and AM fungal inoculation, showing that the function of this gene requires attention. *CsHsp70-8* is known to belong to the group II, which is involved in endoplasmic reticulum metabolism [46]. This suggests that AM fungi resist H stress by activating endoplasmic reticulum metabolism, but more research is needed to unravel the underlying mechanisms. In addition, Rivera-Becerril et al. [45] suggested that *Hsp70s* may maintain the membrane integrity of arbuscule-containing cells under H stress, contributing to high H resistance. This indicates that AM plants did not require up-regulation of a large amount of *CsHsp70s* gene expression, thus implying that mycorrhizal plants did not suffer strong damage under H stress, which is consistent with the results of plant growth changes. Therefore, mycorrhizal cucumbers do not require *CsHsp70s* to initiate H resistance. However, transcriptome sequencing will be used in conjunction with the stress-responsive gene expression patterns to further clarify the molecular mechanisms of mycorrhizal enhancement of H tolerance in cucumbers. It is also necessary to focus on the clarification of the functions of *CsHsp70-8* and *CsPIP1*;6 in arbuscule-containing cortical cells of roots.

## 4. Conclusions

A short-term (80 h) H treatment significantly inhibited cucumber growth, and inoculation with the AM fungus *D. versiformis* mitigated the inhibitory effect. In addition, root arbuscular mycorrhizae significantly increased leaf osmolyte levels, providing a guarantee for mycorrhizal plants to maintain great osmotic regulation under H stress. These results partly confirmed the hypothesis described above, namely, AM fungi could enhance the H tolerance in cucumbers, associated with the increase in osmolytes. Because mycorrhizal cucumber plants were little affected by a short-term H treatment, the expression of many *CsHsp70s* and *CsPIPs* genes was not activated in the leaves. However, deciphering in-depth mycorrhizal thermotolerance mechanisms on host plants using omics techniques has yet to be further investigated, in both the host and the mycorrhizal fungi themselves. AM fungi, such as *D*. *versiformis*, can be introduced into cucumbers during nursery or seedling transplantation to enhance H resistance.

## 5. Materials and Methods

### 5.1. Plant Growth and AM Fungal Inoculation

Seeds of the cucumber variety ‘Pamandi’ were subjected to a 55 °C water bath for 10 min, followed by immersion in a 28 °C water bath for 4 h and placement in Petri dishes at an air temperature of 26 °C for 48 h to induce their germination. The treated seeds were subsequently sown in 32-hole plugs containing autoclaved substrates in an incubator at a day/night temperature of 28 °C/20 °C (16 h/8 h) and a relative humidity of 80%. After a period of two weeks, the cucumber seedlings were transplanted into a plastic pot pre-filled with 1.78 kg of autoclaved soil-sand mixture in a ratio of 3: 1 (*v/v*).

An AM fungus, *Diversispora versiformis*, was introduced into the pot at the time of cucumber transplanting, with 32 spores/g inoculum. The AM fungal inoculation treatment (+*Dv*) involved the application of 120 g of AM fungal inoculum plus 2 mL of inoculum filtrates (25 µm size) to a plastic pot. However, the non-AM fungal treatment (−*Dv*) received the same amount of autoclaved AM fungal inoculum.

After AM fungal inoculation, the seedlings were grown in an environment described by Tian et al. [47], with a day/night temperature of 25 °C/18 °C (16 h/8 h). Forty days later, half of the +*Dv* and −*Dv* seedlings were still under the above-mentioned environmental conditions, which was designated as no heat stress (NH). Nonetheless, the other half was transferred to a heat-stressed (H) environment with 38 °C/30 °C (16 h/8 h; day/night temperatures) for a short-term (80 h) treatment. Such H treatment consisted of a day temperature of 38 °C for 56 h and a night temperature of 30 °C for 24 h. All the seedlings were then harvested.

### 5.2. Experimental Design

The experiment consisted of two AM fungal treatments (+*Dv* and −*Dv*) and two temperature conditions (NH and H). Therefore, there were four treatments in total: NH + *Dv*, NH − *Dv*, H + *Dv*, and H − *Dv*, each with five replicates.

### 5.3. Variable Measurements

The height and stem diameter of the treated plants were measured on the day of harvest. The chlorophyll index in the second fully expanded leaf at the tip was measured using a portable polyphenol-chlorophyll meter (Dualex Scientific^+^, Force-A, Paris, France). The plants were then removed from the pots. The soil adhering to the root surface was washed, and the biomass of the plants was weighed.

Root mycorrhizal staining was performed using the protocol outlined by Phillips and Hayman [48], and the mycorrhizal colonization rate was defined as the percentage of colonized root segment length in total root length.

The concentrations of glucose, fructose, and sucrose in leaves were carried out using the method described by Wu et al. [49], where both sucrose and fructose were determined using the resorcinol (0.1%) colorimetric reaction with sucrose and fructose as standard curves, and glucose was measured by the *o*-dianisidine dihydrochloride (1 mg/mL) reaction with glucose as standard curves.

Leaf proline concentrations were determined by the acidic ninhydrin method [50]. Leaf samples (0.3 g) were mixed with 5 mL of 3% sulphosalicylic acid at 100 °C for 10 min. After filtration, 1 mL of the filtrate was reacted with 1 mL of glacial acetic acid and 1 mL of 25 mg/mL acidic ninhydrin solution at 100 °C for 30 min. After cooling, 2 mL of toluene was added and mixed well. The upper layer was centrifuged at 3000× *g* for 5 min and the absorbance at 520 nm was measured using toluene as a blank and proline as standard curves.

Leaf betaine concentrations were assayed using the method described by Wu et al. [51]. Dry leaf samples (0.25 g) were treated with 10 mL of distilled water for 3 h. After filtration, the filtrate was adjusted to pH 1.0 ± 0.1 using hydrochloric acid, and the volume was fixed to 25 mL. The supernatant was added to 1.5 mL of 99% ether solution and centrifuged at 10,000× *g* for 15 min. The supernatant was then placed in a ventilated area for 10 h. The 5 mL of 70% acetone solution was added and the absorbance was measured at 525 nm, using betaine as the standard sample.

A Plant RNA Rapid Extraction Kit (Aidlab) was utilized to extract total RNA from the leaves. After checking for the integrity and purity, the acquired RNA was reverse-transcribed into cDNA with the PrimeScript™ RT reagent Kit with gDNA Eraser (TaKaRa). Specific primers (Supplementary Material Table S1) were constructed using Primer Premier 5.0 software for qRT-PCR analysis of the 11 *CsHsp70* homologs and 8 *CsPIPs* homologs derived from the wide-genome of cucumbers and Genome Database for Rosaceae. The ubiquitin extension protein (UBI-ep) was chosen as the reference gene [52]. The qRT-PCR system consisted of 10 μL SYBRGREEN PCR Master Mix, 6.4 μL ddH_2_O, 2 μL cDNA, and 0.8 μL each of the forward and reverse primers, respectively. The Bio-Rad CFX system was used for qRT-PCR under the condition reported by Tian et al. [48], with relative gene expression calculated using the 2^−ΔΔCT^ method [53] and the NH-*Dv* treatment served as the control.

### 5.4. Statistical Analysis

The SAS8.0 software was used to conduct the ANOVA on the obtained data, with the use of Duncan’s new multiple range test to analyze significant (*p* < 0.05) differences between treatments.

## Figures and Tables

**Table 1:** AM fungal effects on mycorrhizal colonization and growth behavior of cucumbers under no heat stress and heat stress conditions.

Treatments	Root AM Fungal Colonization (%)	Plant Height (cm)	Stem Diameter (mm)	Biomass (g/Plant)
NH + *Dv*	64.10 ± 4.67 a	33.0 ± 5.1 a	6.78 ± 0.48 a	15.08 ± 1.27 a
NH − *Dv*	0 c	12.3 ± 1.3 c	4.18 ± 0.77 c	4.93 ± 0.43 c
H + *Dv*	47.31 ± 9.12 b	26.7 ± 3.7 b	5.75 ± 0.56 b	13.45 ± 1.92 b
H − *Dv*	0 c	7.2 ± 0.4 d	3.42 ± 0.22d	2.73 ± 0.20 d

Data (means ± SD, *n* = 5) followed by different letters indicate significant (*p* < 0.05) differences among treatments. Abbreviations: NH, no heat stress; H, heat stress; +*Dv*, inoculation with *D. versiformis*; −*Dv*, inoculation without *D. versiformis*.

## Data Availability

All the data supporting the findings of this study are included in this article.

## Supplementary Materials

The following are available online at the following supporting information can be downloaded at: https://www.mdpi.com/article/10.3390/plants12162917/s1, Table S1: Specific primer sequences of selected genes in this study for qRT-PCR.
