# Supplementary material for: Mycorrhizal Fungal Effects on Plant Growth, Osmolytes, and CsHsp70s and CsPIPs Expression in Leaves of Cucumber under a Short-Term Heat Stress
Source: Plants (Basel). 2023 Aug 11;12(16):2917. doi: 10.3390/plants12162917 (PMC10458863; doi:10.3390/plants12162917)
Supplement: Supplementary file 1 [file plants-12-02917-s001.zip › plants-2462193-supplementary.pdf]

Supplementary material Table S1 Specific primer sequences of selected genes in this study for qRT-PCR.

Gene names	Accession number	Primer sequences (5′→3′)
CsPIP1;1	Csa5M199270.1	F: GGGAACACGTGTAAGACCGT
		R: ACACTGCAAATCCGATGGGT
CsPIP1;2	Csa5M198770.1	F: GAGTCAACCGATCTCCGTCC
		R: CCGGATTAATGTGCCCACCT
CsPIP1;5	Csa6M445090.1	F: TGGGTCTTCGGGGGTATGAT
		R: CAAGGCTCGGACCAACGATA
CsPIP1;6	Csa3M739030.1	F: CGCTGGGATTTCTGGTGAGT
		R: AACACGGCAAACCCAATTGG
CsPIP2;1	Csa6M445130.1	F: GCGGCGAGAAATTCACATCC
		R: CAGCTCTCACCAACGACACT
CsPIP2;2	Csa6M445120.1	F: CAGCGGTGACTTTTGGGTTG
		R: CTGGTCATCCCATGGCTTGT
CsPIP2;3	Csa6M445140.1	F: ATCGCCACTCTCCTCTTCCT
		R: AACACGGCAAACCCAATTGG
CsPIP2;6	Csa6M445150.1	F: TTGCTCCCCTATGGCCAAAG
		R: GAGGGGTCGAGAAGAGGTCT
CsHsp70-1	CsaV3_2G009170	F: AAGGAGAGCGAGCAAGAACC
		R: ATCATCACCCATCGGAGCAC
CsHsp70-2	CsaV3_2G011210	F: ATGTCGGAGGTGGATGAGGA
		R: TAATGAAGCGGCAGTGGGTT
CsHsp70-3	CsaV3_3G013380	F: TGACATGCTCTCTGGTGCTG
		R: CTGTGACACCCCTCCTCCTA
CsHsp70-4	CsaV3_3G022450	F: GGAATGACCAAGGCTGAGCT
		R: GCCAACTAAACTCCCCAGCT
CsHsp70-5	CsaV3_4G014660	F: ACGATGCACAGAGACAAGCA
		R: AAGATGCTTTGCGCCACTTG
CsHsp70-6	CsaV3_4G024840	F: GGAACCCGTCGAGAAGTGTT
		R: GTGGTCTTGTCTTCGGCAGA
CsHsp70-7	CsaV3_4G032260	F: GCGTACCAAAATTGCAGGCA
		R: TCCACAGTTGCATTGGACGA
CsHsp70-8	CsaV3_5G001960	F: GACTGCAGGAACCTCGGAAA
		R: ACCTTTGGTAGACTGCGGTG
CsHsp70-10	CsaV3_5G026520	F: TCGGTGATGCTGCCAAGAAT
		R: GGGATCACCTTGAATGGCCA
CsHsp70-11	CsaV3_7G024760	F: ATGGTATCCCTCCTGCACCT
		R: CTAGAGGCTGTTGCTTGGCT
CsHsp70-12	CsaV3_7G033150	F: GCGTTACCTGGGTGAGTTGA
		R: TCAAAGGTGCCACAGGGTTT
UBI-ep	AY372537	F: CACCAAGCCCAAGAAGATC
		R: TAAACCTAATCACCACCAGC
